# Structure and function of African swine fever virus proteins: Current understanding

**DOI:** 10.3389/fmicb.2023.1043129

**Published:** 2023-02-10

**Authors:** Sicheng Yang, Chun Miao, Wei Liu, Guanglei Zhang, Junjun Shao, Huiyun Chang

**Affiliations:** African Swine Fever Regional Laboratory of China (Lanzhou), State Key Laboratory of Veterinary Etiological Biology, Lanzhou Veterinary Research Institute, Chinese Academy of Agricultural Sciences, Lanzhou, Gansu, China

**Keywords:** African swine fever, African swine fever virus, structural proteins, non-structural proteins, capsid protein, inhibitor of apoptosis protein

## Abstract

African swine fever virus (ASFV) is a highly infectious and lethal double-stranded DNA virus that is responsible for African swine fever (ASF). ASFV was first reported in Kenya in 1921. Subsequently, ASFV has spread to countries in Western Europe, Latin America, and Eastern Europe, as well as to China in 2018. ASFV epidemics have caused serious pig industry losses around the world. Since the 1960s, much effort has been devoted to the development of an effective ASF vaccine, including the production of inactivated vaccines, attenuated live vaccines, and subunit vaccines. Progress has been made, but unfortunately, no ASF vaccine has prevented epidemic spread of the virus in pig farms. The complex ASFV structure, comprising a variety of structural and non-structural proteins, has made the development of ASF vaccines difficult. Therefore, it is necessary to fully explore the structure and function of ASFV proteins in order to develop an effective ASF vaccine. In this review, we summarize what is known about the structure and function of ASFV proteins, including the most recently published findings.

## 1. Introduction

African swine fever (ASF), caused by the African swine fever virus (ASFV), is a severe animal disease, with mortality approaching 100% for virulent strains. ASFV, the only known DNA arbovirus (Hakizimana et al., 2020), is also the only member of the ASFV genus of the family *Asfarviridae* (Mebus, 1988).

ASF has existed for over 100 years, and regional outbreaks have been reported (Mighell and Ward, 2021; Penrith and Kivaria, 2022). ASFV was first reported in Kenya in 1921. Subsequently, it spread to countries in Western Europe, Latin America, and Eastern Europe (Gaudreault et al., 2020). By the mid-1990s, ASFV had been eradicated in the Americas and Europe, but Sardinia remained endemic (Costard et al., 2009; Gaudreault et al., 2020). In 2014, ASFV spread into the Russian Federation and Eastern Europe, where it continues to circulate (Vergne et al., 2016). An outbreak of ASF has occurred in Asia. In August 2018, the first ASF case in China was identified in Shenyang, Liaoning Province. Subsequently, outbreaks were reported in other provinces in China, thus causing extensive economic losses (Tao et al., 2020). Currently, the spread of the ASF continues with outbreaks in Southeast Asia including Thailand, the Philippines, and other countries (Cooper et al., 2021; Cadenas-Fernández et al., 2022; Iscaro et al., 2022; Ramirez-Medina et al., 2022a). ASFV infections of wild boar are more serious than infections of domestic pigs (O’Neill et al., 2020; Sauter-Louis et al., 2021). Because no effective ASF vaccine is available, the prevention and control of ASF is primarily at the level of biosecurity, employing multiple lines of defense that block the spread of ASF (Liu et al., 2021). These approaches are less than optimal and improvements in prevention and control of the virus, as well as the development of an ASF vaccine, are essential. It is therefore necessary to identify the structure and function of ASFV proteins.

## 2. A brief description of ASFV

**Figure 1:**
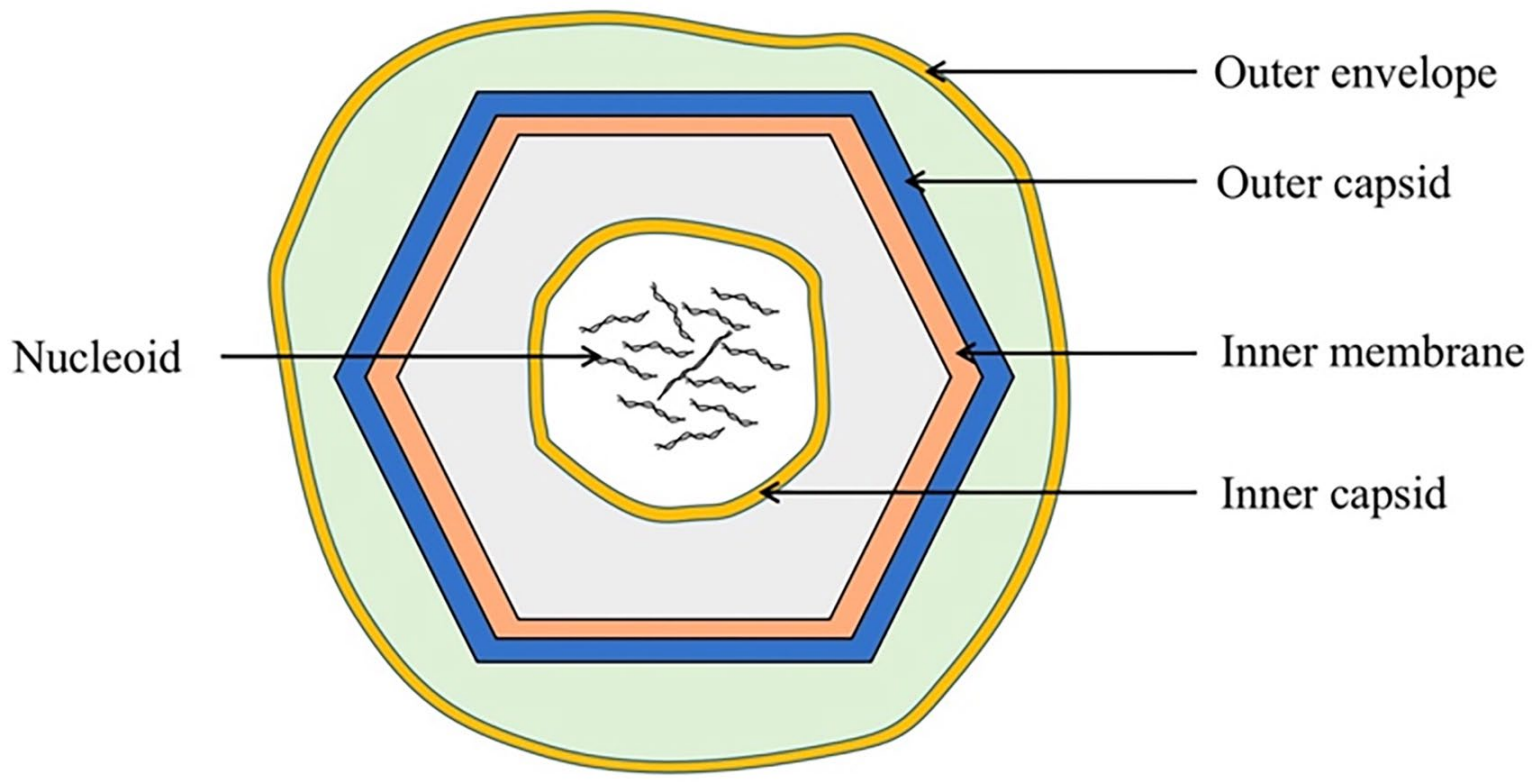
Schematic diagram of the ASFV structure. ASFV is composed of an external envelope, outer capsid, inner membrane, inner capsid, and nucleoid.

ASFV is a double-stranded DNA virus (Hakobyan et al., 2018) with a diameter of approximately 260–300 nm. It includes an envelope, capsid, inner membrane, core shell, and genome (Figure 1; Revilla et al., 2018).

The external envelope, the outermost structure of ASFV, may be involved in viral attachment and endocytosis. ASFV enters host cells through clathrin-mediated endocytosis (CME) and macropinocytosis (Mercer et al., 2010; Galindo et al., 2015; Zhang et al., 2021). CD2v, an envelope protein, is composed of a signal peptide, transmembrane region, and two immunoglobulin-like domains. CD2v has a variable number of proline-rich repeats within the cytoplasmic domain that can interact with the actin-binding adaptor protein, SH3P7, to promote vesicle transport and signal transduction (Goatley and Dixon, 2011; Jia et al., 2017; Alejo et al., 2018).

The capsid has an icosahedral structure and is adjacent to the external envelope membrane, which protects the virus from nucleases or other physical and chemical factors within the environment. The major capsid protein, p72, is the most dominant structural component of the virion and constitutes 31%–33% of the total mass of the virion (Andrés et al., 2020). This major viral protein induces an antibody response after viral infection. Owing to its conservation and immunogenicity, it is widely used as an antibody detection target for ASFV infection (Geng et al., 2022; Wang C. et al., 2022).

The inner membrane contains an icosahedral capsid that encloses the core shell and the nucleoid. Inside the inner envelope are p17, pE183L, p12, pE248R, and pH108R (Alejo et al., 2018). The inner membrane protein, p17, is an essential and highly abundant protein required for the assembly of the capsid and for icosahedral morphogenesis (Liu et al., 2019; Andrés et al., 2020).

The correct assembly of the core shell depends on the assembly of the outer capsid and the inner membrane, but not vice versa. The core shell may serve to confine and protect the viral genome from host nucleases as well as from host dsDNA sensors, inhibiting the innate immune response during initial infection (Andrés et al., 2020).

The viral genome is enclosed by a core shell, approximately 170 to 194 kbp in length, ending in hairpin loops, comprised variable regions that contain tandem repeats and multigene families (Shimmon et al., 2021). After ASFV endocytosis, the viral genome is delivered into the cytosol, wherein transcription of the early viral genes is initiated (Cackett et al., 2020).

The ASFV genome encodes over 60 structural proteins and 100 non-structural proteins (Jia et al., 2017). Of these, approximately 50 genes encode viral structural proteins with known functions (Sun et al., 2021), although the function of many other proteins is unknown. The mechanism of ASFV infection is not completely understood, which makes vaccine development difficult. Structural and functional analysis of these proteins is essential to the future development of antiviral drugs and vaccines. In this review, recent analysis of ASFV protein structure and function is summarized.

## 3. ASFV protein structure and function

### 3.1. Proteins involved in invasion

The entry of ASFV into host cells occurs through receptor-mediated endocytosis; however, the cellular receptors for binding and entry are unknown. Clathrin-mediated endocytosis (CME) or macropinocytosis is the most likely route of endocytosis (Mercer et al., 2010; Galindo et al., 2015; Zhang et al., 2021). This process requires dynein. Viruses use dynein to facilitate internalization and intracellular transport (Hernáez et al., 2010). Previous studies have shown that p54 interacts with dynein and consequently participates in viral invasion (Alonso et al., 2001). Moreover, pE248R participates in membrane fusion, thus promoting ASFV infection; however, the mechanism is currently unclear (Rodríguez et al., 2009). Therefore, the structural analysis of p54 and pE248R is expected to provide a deeper understanding of ASFV invasion.

#### 3.1.1. p54

p54, encoded by the E183L gene, is a transmembrane protein located on the external envelope (Rodriguez et al., 1994; Rodríguez et al., 2004). It contains a putative transmembrane domain and disulfide-linked homodimers (Rodríguez et al., 2004). The 13-amino-acid domain of p54 binds LC8, which is part of the microtubular motor complex, and subsequently hijacks the microtubule motor complex cellular transport machinery (Alonso et al., 2001). After this binding, p54 transports the virus intracellularly along microtubules. The virus rapidly reaches the perinuclear area *via* transport through the cytosol. Subsequently, viral replication occurs. In this process, the disruption of the function of the microtubule motor dynein inhibits the transport of viruses (Hernáez et al., 2010). This interaction is critical during viral internalization and transport to factory sites (Hernáez et al., 2004). In addition, the interaction of the microtubule motor protein LC8 with p54 protein may allow viruses to use host cells to promote viral transport and replication (Alonso et al., 2001). However, owing to the complexity of the system, the mechanism of this interaction remains elusive (Hernáez et al., 2010). Therefore, analysis of the structure of p54 may facilitate understanding of this mechanism of interaction, and enable blocking of the critical early stage of ASFV infection.

#### 3.1.2. pE248R

pE248R, encoded by the E248R gene, is localized at the inner envelope and contains intramolecular disulfide bonds, a putative myristoylation site, and a hydrophobic transmembrane region near its carboxy terminus (Rodríguez et al., 2009). pE248R is associated with the infectivity of ASFV. A lack of pE248R decreases the infectivity of ASFV. In addition, pE248R is required for cell entry and membrane fusion (Rodríguez et al., 2009). pE248R shows high amino acid sequence similarity with VACV protein L1—a component of the poxviral multiprotein entry/fusion complex, which is required for proper membrane fusion and core penetration (Moss, 2012; Hernáez et al., 2016). Membrane fusion may occur during the passage of virions through the cytoplasmic vacuolar membrane and subsequently deliver viral “cores” into the cytoplasm (Valdeira et al., 1998; Rodríguez et al., 2009). In addition, ASFV entry relies on a fusion machinery comprising pE248R and pE199L (Matamoros et al., 2020). Further studies on this protein and fusion machinery may reveal the mechanisms of ASFV entry and postentry pathways.

### 3.2. Proteins involved in replication and transcription

ASFV replication occurs mainly in the cytoplasm 6–8 h after host cell infection. The expression of intermediate and late genes, which encode viral particle structure-related proteins and early transcription factors, starts after 8–16 h (Gaudreault et al., 2020; Wang Y. et al., 2021). The proteins currently known to be involved in ASFV replication and transcription are shown in Table 1.

**Table 1:** Proteins involved in the replication and transcription of ASFV.

Involved in replication		Involved in transcription	
Proteins	Function	Proteins	Function
p37	Involved in the export of the viral genome from the nucleus to the cytoplasm in infected cells after the brief nuclear replication phase (Eulálio et al., 2004).	p15	Involved in viral transcription (Guo et al., 2021).
p14	Involved in the formation of a complex with viral DNA, facilitation of its entry into the nucleus, and subsequent initiation of DNA replication (Eulálio et al., 2004).	pB263R	Involved in TATA binding function and viral gene transcription (Kinyanyi et al., 2018).
p10	Involved in the late phase of the viral replication cycle (Nunes-Correia et al., 2008).	pQP509L	Assist termination and release of late viral transcripts (Freitas et al., 2019).
pA104R	Involved in viral genome packing and replication (Urbano and Ferreira, 2020).	pQ706L	Regulates elongation and release of late viral transcripts (Freitas et al., 2019).
pP1192R	Involved in the replication of viral genome (Coelho and Leitão, 2020).		
I73R	Involved in nuclear organization during the initial phase of ASFV replication (Sun et al., 2022).		

#### 3.2.1. pS273R catalyzes the pp220 polyprotein precursors into p14 and p37 that are involved in replication

**Figure 2:**
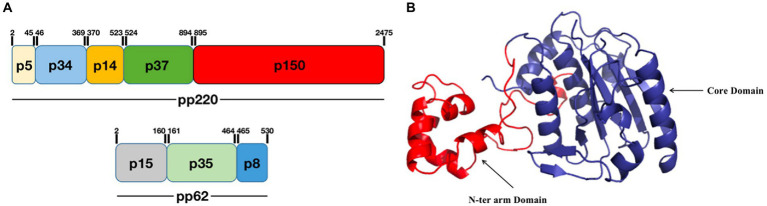
Schematic diagram of the ASFV pS273R structure. **(A)** pS273R catalyzes the hydrolysis of pp220 to p5, p34, p14, p37, and p150, and pp62 to p15, p35, and p8. **(B)** The pS273R protein consists of an “arm domain” (red) and a “core domain” (dark blue; Li et al., 2020).

pS273R is a specific SUMO-1 cysteine protease that catalyzes the maturation of the pp220 and pp62 polyprotein precursors into the core-shell proteins p5, p34, p14, p37, p150, p15, p35, and p8 (Figure 2A; Andrés et al., 2002a; Alejo et al., 2018). The pS273R protease comprises 12e α-helices, seven β-strands, and one 3_10_-helix, which fold into two close but distinct domains: the core domain and arm domain (Figure 2B). The core domain consists of a sandwich-like fold structure that contains residues N84 to A273 and shares high structural similarity with chlamydial deubiquitinating enzyme, sentrin-specific protease, and adenovirus protease. The arm domain is unique to ASFV, and consists of five α-helices (α1 to α5) and one 3_10_ helix; residues M1 to N83 of pS273R are necessary for enzymatic activity. The pS273R protease has a catalytic triad, C232-H168-N187, which catalyzes ubiquitin or ubiquitin-like proteins such as ISG15 or SUMO (Báez-Santos et al., 2015; Li et al., 2020). The pS273R protease may antagonize the host IFN-I pathway by deubiquitinating specific proteins (Li et al., 2020). Furthermore, targeted inhibitors have been designed for pS273R and include tetrapeptide nitrile compounds (Mac Sweeney et al., 2014; Grosche et al., 2015; Li et al., 2020). Evaluation of the biochemical activity of pS273R has demonstrated that these inhibitors bind the enzyme’s active site, thus providing a reference for the design of antiviral drugs based on the ASFV protein structure.

pp220, encoded by the CP2475L gene, is an N-myristoylated precursor polypeptide that may function as a membrane-anchoring signal that facilitates the binding of the developing core shell to the inner envelope (Andrés et al., 2002a,b). pp220 is essential for core assembly rather than the construction of external viral domains; moreover, it is directly or indirectly involved in the incorporation of other major core components, including the viral genome, into virus particles (Andrés et al., 2002a,b). The processing of pp220 is dependent on the expression of p72. Unprocessed pp220 assembles into aberrant zipper-like elements consisting of an elongated membrane-bound protein structure by the repression of p72 synthesis (Andrés et al., 2002a).

p37 and p14 from the proteolytic processing of pp220 are both core shell proteins (Andrés et al., 2002a). p37, the first nucleocytoplasmic shuttling protein encoded by the ASFV genome, is involved in the nuclear transport of viral DNA during the ASFV replication cycle (Eulálio et al., 2004). Studies have shown that p37 is both imported into the nucleus and exported from the nucleus to the cytoplasm through a process mediated by CRM-1 receptors (Eulálio et al., 2007). p14 is also a nucleocytoplasmic shuttling protein. However, unlike p37, it only enters the nucleus but is not exported to the cytoplasm. p37 and p14 may be involved in the formation of a complex with viral DNA, thus facilitating entry into the nucleus and subsequent initiation of DNA replication (Eulálio et al., 2004).

#### 3.2.2. The viral nucleoid protein p10 and pA104R involved in replication

p10, encoded by the K78R gene, is located in the viral nucleoid and has been shown to exhibit DNA-binding activities that may be related to the characteristic helix-turn-helix structural motif (Nunes-Correia et al., 2008). Both the C-terminal helix rich in lysine residues and the serine-rich residues found in the N-terminal helix of p10 are crucial for the interaction with dsDNA. p10 exploits cellular mechanisms to achieve active nuclear import (Istrate et al., 2022). In ASFV-infected cells, p10 protein strongly accumulates in the nucleus in late stages post-infection and therefore may perform an important function in the nucleus during the late phase of the viral replication cycle (Nunes-Correia et al., 2008). The crystal structure of p10 has not yet been reported. Thus, further study of the structure of p10 may aid in understanding of the binding of p10 to DNA in the viral life cycle, and provide a theoretical basis for the development of an ASF vaccine.

**Figure 3:**
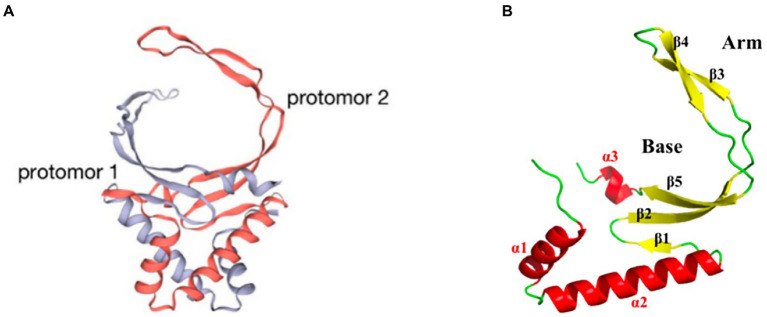
Schematic diagram of the ASFV pA104R structure. **(A)** The dimer structure of the pA104R protein. Gray: protomer 1; light red: protomer 2. **(B)** Distribution of α-helices and β-sheets in the pA104R protomer.

pA104R is a homodimer with two domains: an α-helical region (AHR) and a β-strand DNA-binding region (BDR; Figure 3A). The secondary structural elements of the protomer of the pA104R dimer include two α-helixes (α1 and α2) followed by five β-sheets (β1-5), which end with a final α/3_10_-helix (α3/η1). The β1, β2, and β5 strands form an antiparallel β-sheet, which constitutes the bottom of the BDR, whereas the β3 and β4 strands compose the arms of the BDR (Figure 3B; Liu et al., 2020). The positively charged amino acids K92, R94, K97, R69, H72, K83, and K85 are densely distributed within the BDR base and the BDR arm regions, and serve as potential binding sites for the negatively charged DNA backbone. Residues in the BDR base region are important contributors to DNA interaction (Liu et al., 2020; Urbano and Ferreira, 2020). pA104R has been suggested to bind dsDNA and ssDNA, with a higher affinity for the former. Furthermore, stilbene derivative compounds bind pA104R and inhibit the pA104R-DNA interaction, thus preventing ASFV replication (Liu et al., 2020). pA104R also participates in the modulation of viral DNA topology and genome packaging, and therefore is likely to be involved in viral DNA replication, transcription, and packaging (Frouco et al., 2017). ASFV DNA packaging is a highly dynamic process, and how pA104R and DNA participate in the process is currently unclear. Future studies on pA104R are expected to provide a deeper understanding of ASFV DNA replication and transcription.

#### 3.2.3. pP1192R involved in replication

pP1192R is a type II DNA topoisomerase (topoII) encoded by ASFV ORF P1192R, which is located in cytoplasm and accumulating in the viral factories (Coelho et al., 2015). TopoIIs are able to relax supercoils, to resolve knots and tangles in the DNA (Nitiss, 2009). Thus, pP1192R may play an active role in the replication of ASFV genome. And pP1192R can alleviate the topological tension that generated by the binding of pA104R to the DNA (Coelho and Leitão, 2020). In addition, structural analysis of pP1192R and studies with known structures of topoII inhibitors may lead to the discovery of anti-ASFV drugs (Constantinou et al., 1995; Mottola et al., 2013), which can eventually counteract the activity of the viral topoisomerase without hindering the cellular homologs (Coelho and Leitão, 2020).

#### 3.2.4. I73R Protein involved in replication

ASFV encodes a 72-amino acid protein, I73R protein (Cackett et al., 2020). I73R protein is expressed in the nucleus in early stages post-infection and gradually translocates from the nucleus to the cytoplasm. This protein has no homology with any known or other functionally identified protein sequence. I73R protein has a classical α/β architecture wherein three α-helices form the core of the domain, and helices 2 and 3 form the helix-turn-helix unit (Sun et al., 2022). I73R protein shares structural similarity with Z-DNA-binding domains (Zα), which are specific for the left-handed conformation of nucleic acid duplexes of DNA, DNA/RNA hybrids, and RNA (Lee et al., 2016). I73R protein also binds CpG repeats DNA duplexes with a high propensity for forming Z-DNA during DNA-binding assays. Key residues of I73R protein are highly conserved in other Zα domain proteins, and behave similarly to those of other Zα domain-containing proteins such as ADAR1, PKZ, and DAI (Ha et al., 2008, 2009; de Rosa et al., 2013). As a member of the Zα family with the ability to bind Z-DNA, I73R protein may be involved in the process of immune evasion (Sun et al., 2022). I73R protein has also been shown to inhibit host gene expression and to play an important role in host-pathogen interactions and nuclear organization during the initial phase of ASFV replication (Sun et al., 2022). However, the mechanism through which I73R protein promotes ASFV infection is unclear, and thus further research is necessary.

#### 3.2.5. p15 involved in transcription

**Figure 4:**
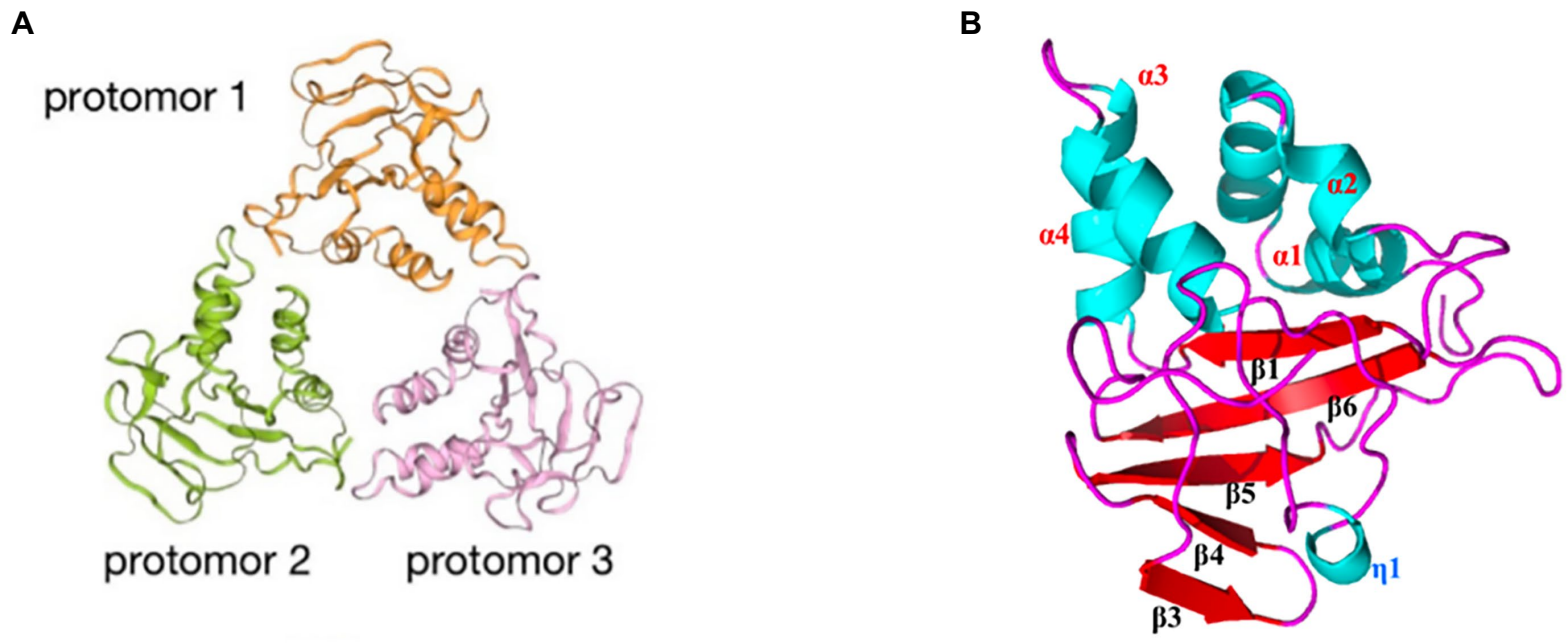
Schematic diagram of the ASFV p15 structure. **(A)** The trimeric structure of the p15 protein. Orange: protomer 1; green: protomer 2; pink: protomer 3. **(B)** Distribution of α-helices and β-sheets in the p15 proteome.

ASFV p15 is one of the mature products derived from polyprotein pp62 processing at the Gly-Gly-Xaa sequence (Simón-Mateo et al., 1997). The viral polyprotein processing proteinase pS273R plays a key role in this process (Li et al., 2020). p15 exists as several types of oligomers such as dimers and trimers. The dimer (Figure 4A) of p15 arises from disulfide bond formation between the Cys9 and Cys30 residues (Guo et al., 2021). These disulfide bonds are critical for the formation of the p15 structure. The structure of the p15 protomer consists of four α-helixes and six β-sheets (Figure 4B). p15 has been suggested to bind nucleic acids, and to have a stronger affinity for ssDNA than dsDNA. Lys10 and Lys39 are essential for the affinity of DNA binding (Guo et al., 2021; Wang G. et al., 2021). p15’s binding to ssDNA suggests its potential involvement in viral transcription and DNA replication during the ssDNA phase of the viral life cycle (Guo et al., 2021).

#### 3.2.6. pQP509L and pQ706L involved in transcription

pQP509L and pQ706L are both the SF2 RNA helicases that expressed by ASFV (Freitas et al., 2019). pQP509L was detected from 12 h post infection (hpi) within viral factories and host nucleus, and able to assist termination and release of late viral transcripts, which involved in viral transcription. At later times of infection, it can be also related to the modulation of antiviral responses (Freitas et al., 2019). In addition, pQP509L may be associated with ASFV virulence. The deletion of the QP509L and QP383R genes from ASFV is highly attenuated (Li et al., 2022). But subsequent studies have shown that deleting QP509L gene from the highly virulent Georgia 2010 strain is unable to affect the replication or virulence of ASFV (Ramirez-Medina et al., 2022b). pQ706L was detected only at viral factories from 12 hpi onward, and regulates elongation and release of late viral transcripts to involve the transcription of ASFV (Freitas et al., 2019). However, the mechanism of pQP509L and pQ706L is still unclear. Therefore, the research on their structures and functions is expected to enable us to understand these proteins in depth and is crucial for the ASF vaccine design and responding to emerging ASFV strains.

### 3.3. pNP868R involved in post-transcriptional modification

**Figure 5:**
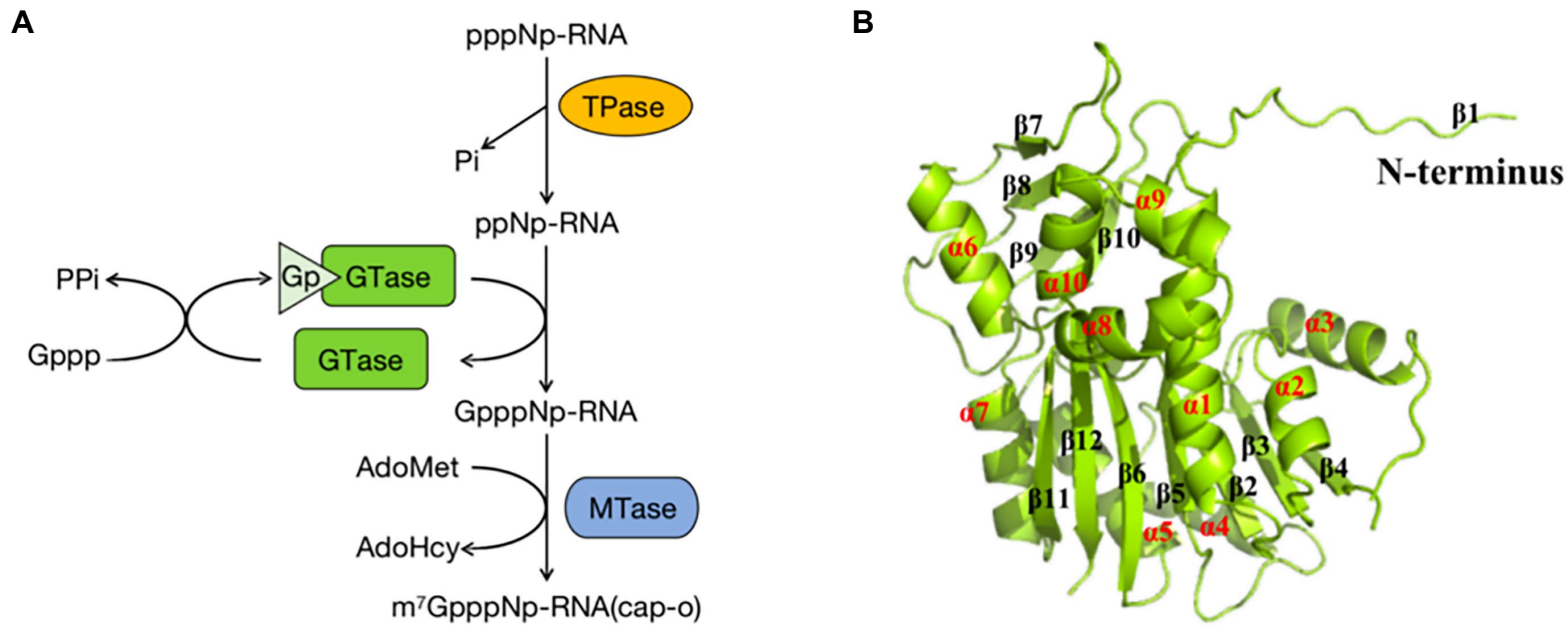
**(A)** The capping mechanism of mRNA involved in pNP868R. First, the triphosphatase (TPase) hydrolyses the γ-phosphate of the nascent RNA (pppNp-RNA, in which N denotes the first transcribed nucleotide and p denotes a phosphate group) to yield a diphosphate RNA (ppNp-RNA) and inorganic phosphate (Pi). Then, guanylyltransferase (GTase) reacts with the α-phosphate of GTP (Gppp), releasing pyrophosphate (PPi) and forming a covalent enzyme–guanylate intermediate (Gp–GTase). The GTase then transfers the GMP molecule (Gp) to the 5′-diphosphate RNA to create GpppNp-RNA. In the final step (guanine-N7)-methyltransferase (N7MTase) transfers the methyl group from S-adenosyl-l-methionine (AdoMet) to the cap guanine to form the cap-0 structure, with the 7-methyl-GpppNp (m7GpppNp) releasing S-adenosyl-l-homocysteine (AdoHcy) as a by-product (Decroly et al., 2011). **(B)** Schematic diagram of the ASFV pNP868R protein structure, showing α-helices and β-sheets within the structure (Du et al., 2020).

pNP868R consists of an N-terminal triphosphatase (TPase) domain, a central guanylyltransferase (GTase) domain, and a C-terminal methyltransferase (MTase) domain. The TPase and GTase domains are members of the triphosphate tunnel metalloenzyme (TTM) and covalent nucleotidyltransferase (NTase) superfamilies, respectively (Pena et al., 1993). The MTase domain exhibits the characteristic core fold of class I family of S-adenosyl-l-methionine (AdoMet)-dependent MTases (Cong and Shuman, 1995). The 5′-triphosphate end of the pre-mRNA is first hydrolyzed to a diphosphate by RNA 5′-TPase. The diphosphate RNA is then capped with GMP by RNA GTase forming GpppRNA. Finally, the GpppRNA cap is converted to a 7-methylguanosine RNA cap (m7GpppRNA) by RNA (guanine-N7)-MTase (Figure 5A; Shuman, 2001; Decroly et al., 2011). The process of mRNA 5′-end capping involved by pNP868R contributes to mRNA stability and efficient translation (Du et al., 2020).

The MTase domain of pNP868R has been resolved and displays a typical class I MTase fold comprised a seven-strand β-sheet (described as β4, β3, α2, β5, β6, β12, and β11) with six helices (α1, α2, α3, α4, α5, and α7). Based on these structures, there is an N-terminal extended β-sheet (β1), followed by a 9-residue β1-α1 loop, and a flap domain between β6 and α7 that contains a four-strand antiparallel β-sheet (β7, β8, β9, and β10), which is flanked by four extra helices (α6, α8, α9, and α10; Figure 5B). The N-terminal extension contains R592 and R597 residues that may be close to the active site upon substrate binding, playing an essential role in substrate recognition as judged by *in vitro* MTase analysis (Du et al., 2020). Thus, the MTase domain plays a particularly important role in the function of pNP868R.

Future analysis of pNP868R substrate recognition and catalysis are expected to provide a direction for the development of effective biologics targeting ASFV-related enzymes.

### 3.4. Proteins involved in ASFV translation

ASFV has self-sufficient replication and transcription mechanisms, but relies on the host translational mechanism for protein synthesis (Zhong et al., 2022). ASFV regulates the DNA translation pathway in a variety of ways, and its products undergo post-translational modifications (Wang Y. et al., 2021). Previous studies have shown that g5Rp, pI215L and pDP71L participate in these processes (Zhang et al., 2010; Dixon et al., 2017; Freitas et al., 2018; Yang et al., 2022), and the structure of g5Rp has been resolved.

#### 3.4.1. g5Rp

**Figure 6:**
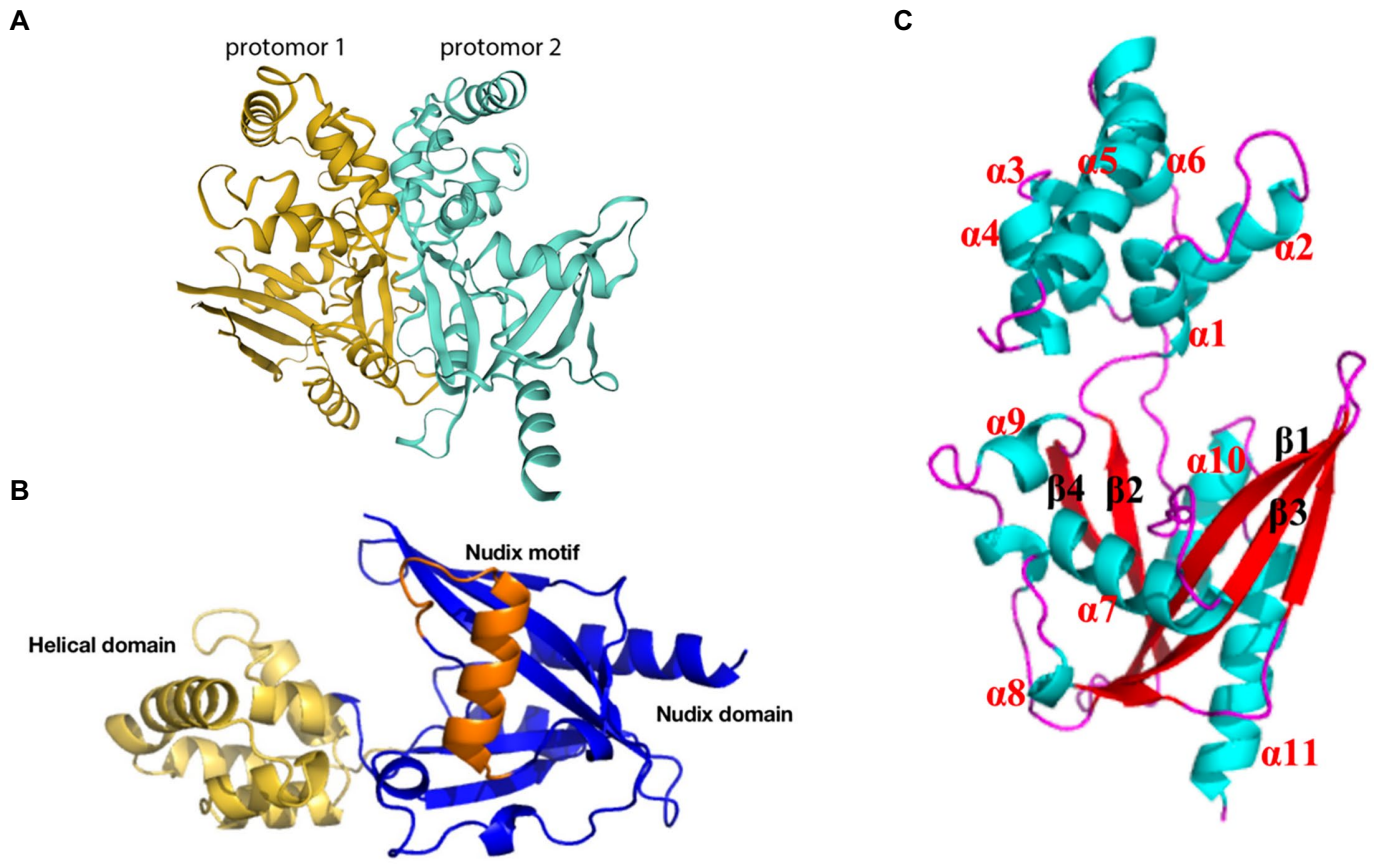
Schematic diagram of ASFV g5Rp structure. **(A)** g5Rp consists of two protomers “back to back,” protomer 1 is in dark yellow and protomer 2 is in cyan (Yang et al., 2022). **(B)** Nudix domain (dark blue), the helical domain (yellow), and the Nudix motif of g5Rp (orange). **(C)** The α-helices and β-sheets of the g5Rp protomer.

g5Rp, the only viral mRNA-decapping enzyme, is expressed in the endoplasmic reticulum at the earliest stage of infection and accumulates throughout the ASFV infection process; this enzyme plays an essential role in the machinery assembly of mRNA regulation and translation initiation. g5Rp forms a stable symmetric dimer of g5Rp crystal packaging, with the dimer composed of two protomers (Figure 6A). Each protomer is composed of a unique N-terminal helical domain and a C-terminal classic Nudix domain (Figure 6B). The helical domain forms a globin-fold-like feature composed of six α-helices (α1 to α6) that connects to the Nudix domain by two hinge linkers. The Nudix domain consists of a central curved β-sheet (β1, β2, β3, β4) surrounded by five α-helices (α7 to α11) and several loops, thereby forming a classic α-β-α sandwich structure (Figure 6C). The helical domain is the major mediator of RNA interaction, with positively charged regions likely contributing to g5Rp binding with RNA (Yang et al., 2022). The g5Rp Nudix domain interacts with RNA, with the g5Rp C-terminal exhibiting substrate selectivity at the RNA binding step (Yang et al., 2022). K8, K95, K133, and R221 are the key residues that mediate g5Rp-RNA interaction and are also important in g5Rp-related cellular RNA degradation *in vivo* (Yang et al., 2022). g5Rp is the only Nudix hydrolase that targets mRNAs of the host cell. After g5Rp binds to RNA of the host cell, it robustly cleaves the mRNA 5′ cap attached to an RNA moiety dependent upon the Nudix motif (Parrish et al., 2009; Yang et al., 2022). g5Rp has a broader range of nucleotide substrate specificities, including a variety of guanine and adenine nucleotides and dinucleotide polyphosphates (Cartwright et al., 2002). Removal of the 5′ cap on host cellular mRNAs by g5Rp is beneficial to viral gene expression during the early stages of infection (Dixon et al., 2013; Sánchez et al., 2013). As originally described, g5Rp dephosphorylates 5-PP-InsP5 (InsP7) to produce InsP6. InsP6 inhibits the mRNA-decapping activity of g5Rp by competing for the substrate mRNA-binding surface of g5Rp.

Although the crystal structure of g5Rp has been resolved, the complex structure formed by the interaction between g5Rp and mRNA is unclear. Therefore, the future resolution of this issue is expected to deepen understanding of the structural basis for g5Rp protease activity, and may have far-reaching significance for the development of new drugs and vaccines against ASFV.

#### 3.4.2. pI215L

pI215L, encoded by ASFV I215L gene, localizes in viral factories and host cell nucleus (Freitas et al., 2018), and the only known virus-encoded E2-ubiquitin conjugating enzyme (UBCv1; Barrado-Gil et al., 2020). UBCv1 has high activity and has a catalytic domain that could associate with several classes of polyubiquitin chains. And the catalytic site Cys85 in its catalytic domain plays an important functional role (Freitas et al., 2018; Barrado-Gil et al., 2020). UBCv1 interact with the 40s ribosomal protein S23 (RPS23), which prevents potential mRNAs or other factors from binding to it to influence the translation (Barrado-Gil et al., 2020). UBCv1 is also able to bind to the eukaryotic initiation factor 4E (eIF4E) and induces this factor overexpression, which result in increased protein synthesis (Barrado-Gil et al., 2020; Romagnoli et al., 2021). Besides, the UBCv1 could bind to the E3 ligase Cullin4B (Cul4B) that has an important role in regulating TSC2 and mTOR signaling and degrading 4E-BP2 eukaryotic translation initiation factor (Wang et al., 2013; Kouloulia et al., 2019; Barrado-Gil et al., 2020). The further study on the structure and function of pI215L is expected to the potential use in the drug against ASF.

#### 3.4.3. pDP71L

pDP71L exists in either a short form of 70 to 72 amino acids or a long form of approximately 184 amino acids in all ASFV isolates, and is expressed late during the replication cycle (Zhang et al., 2010). It binds all three isoforms (α, β, and γ) of the protein phosphatase 1 catalytic subunit (PP1c) (Rivera et al., 2007; Zhang et al., 2010), and the residues V16 and F18A in pDP71L were critical for binding to PP1c (Barber et al., 2017). DP71L decreases the amount of phosphorylated eIF2α by recruiting PP1c, which dephosphorylates eIF2α, and also inhibits the induction of ATF4 and its downstream target CHOP (Zhang et al., 2010). The phosphorylation of eIF2α is a key rate-limiting step in the control of protein synthesis; therefore, the virus can affect protein expression by decreasing the phosphorylation of eIF2α. And CHOP is essential in causing apoptosis in cells with irrecoverable ER stress (Marciniak et al., 2004; Zhang et al., 2010). Thus, pDP71L inhibits apoptosis by inhibiting CHOP induction and activation (Dixon et al., 2017). However, DP71L is not the only factor required by the virus to control the phosphorylation level of eIF2α during infection (Zhang et al., 2010). Therefore, further studies on the mechanisms affecting the phosphorylation level of eIF2α and the synthesis of protein would deepen understanding of the translation process of ASFV.

### 3.5. A224L and A179L proteins inhibit the apoptosis of host cells and consequently promote viral proliferation

#### 3.5.1. A224L protein

A224L protein, a late protein in the viral cycle, is a member of the inhibitor of apoptosis (IAP) family that contains a single BIR motif at the NH2 terminus as well as a sequence that may constitute a zinc finger domain of the 4-cysteine type at the C-terminal region. BIRs are involved in all known interactions between IAPs and other proteins and are required for the antideath activity of IAPs (Deveraux et al., 1997; Harvey et al., 1997). However, A224L protein differs in that it lacks the typical RING motif found in other IAP proteins. Therefore, A224L protein may not have ubiquitin ligase activity (Nogal et al., 2001). It has been suggested that A224L protein promotes cell survival. A224L protein substantially inhibits caspase 3 activity and cell death induced by treatment with tumor necrosis factor α (Nogal et al., 2001). The means by which A224L inhibits cell death was suggested from studies indicating that transient expression of A224L protein activates the NF-kB-dependent reporter C-rel. This NF-kB inducing activity was abrogated by an IKK-2-dominant negative mutant and enhanced by expression of TNF receptor-associated factor 2 (TNF-R2) (Rodríguez et al., 2002). The activation of NF-kB mediated by TNF-R2 can inhibit apoptotic cell death by activating transcription of a number of anti-apoptotic genes including IAP and Bcl-2 family members (Rodríguez et al., 2002; Dixon et al., 2017).

#### 3.5.2. A179L protein

A179L protein, similarly to pDP71L and A224L proteins, suppresses ASFV infection-induced apoptosis. However, among these proteins, only the crystal structure of A179L protein has been solved (Nogal et al., 2001; Banjara et al., 2017; Dixon et al., 2017).

**Figure 7:**
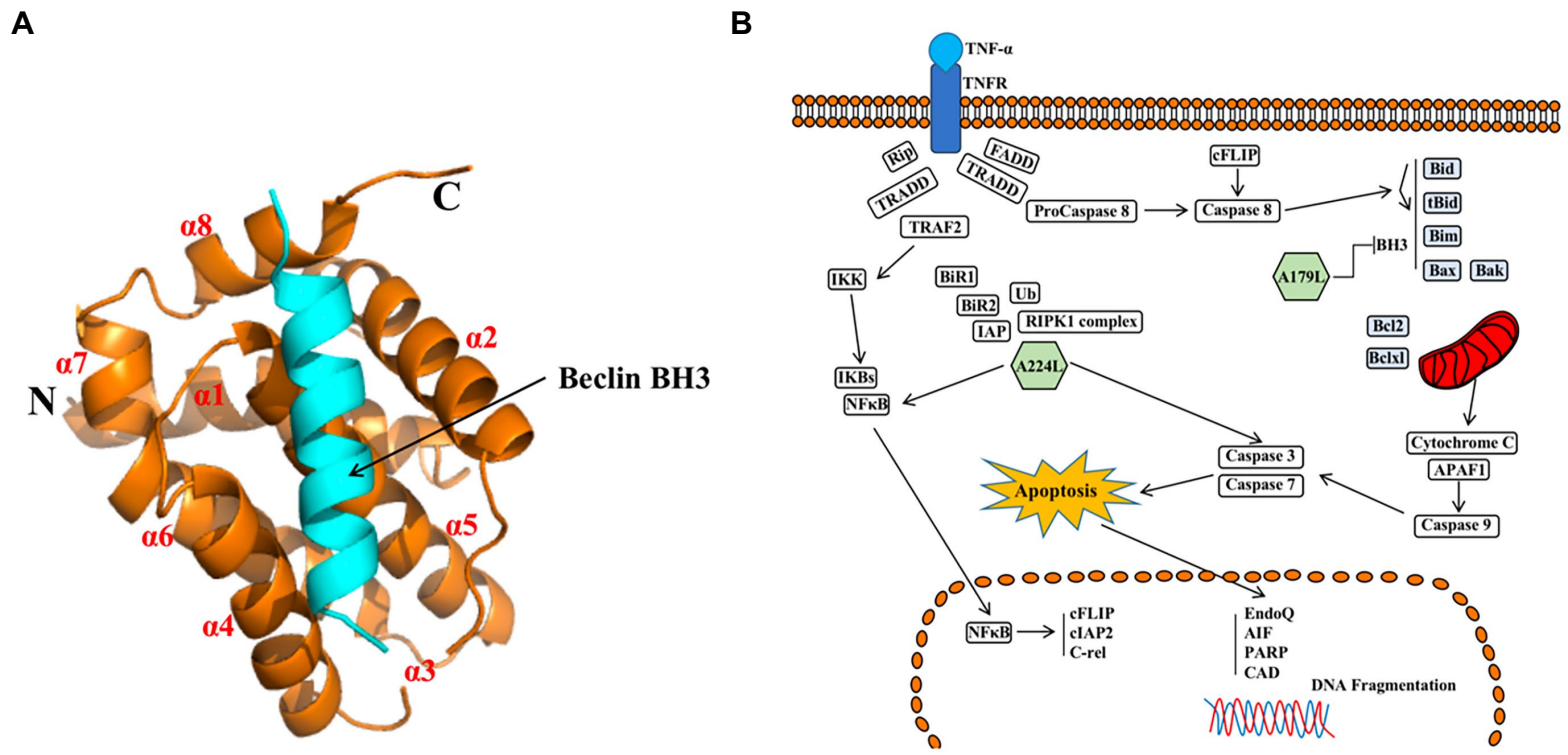
**(A)** Schematic diagram of ASFV A179L and BH3 complex structures (Banjara et al., 2019). **(B)** A224L and A179L of ASFV inhibit apoptosis of infected cells (Dixon et al., 2017). ASFV A224L and A179L are shown as green hexagons. ASFV A179L Bcl-2 family proteins bind to and inhibit several pro-apoptotic proteins with a BH3 motif. A224L IAP family proteins bind to and inhibit caspase 3 and activate the nuclear factor Kappa-light chain enhancer of activated B cell (NF-κB) signaling, thereby increasing the expression of anti-apoptotic genes including cFLIP, cIAP2, and C-rel (Dixon et al., 2017).

A179L protein, a potent inhibitor of apoptosis, is a member of the Bcl-2 family (Brun et al., 1996; Galindo et al., 2008), which is localized in mitochondria and the endoplasmic reticulum (Banjara et al., 2017). A179L protein adopts a Bcl-2 fold featuring eight α-helices arranged in a globular helical bundle fold. The canonical ligand-binding groove is formed by α-helices 2–5 and engages the BH3 motif of the pro-apoptotic protein Bcl-2 (Kvansakul and Hinds, 2013; Banjara et al., 2017, 2019; Figure 7A). Ionic interactions formed by binding of the E76 residue of A179L protein to the Bid R81 ligand, the D80 residue of A179L protein to the Bax K64 ligand, and the K79 residue of A179L protein to the Bax E61 ligand demonstrate the specificity of A179L protein (Banjara et al., 2017). In addition, A179L protein inhibits apoptosis signaling by binding the porcine pro-apoptotic Bcl-2 proteins Bax, Bak, Bim, Bid, Bad, Bik, Bmf, Hrk, Noxa, and Puma (Banjara et al., 2019). A179L protein also binds full length the autophagy regulator Beclin as well as its BH3 motif, thus demonstrating the ability of A179L protein to interfere with both host apoptosis and autophagy signaling (Revilla et al., 1997; Banjara et al., 2017, 2019).

In conclusion, A224L and A179L proteins suppress ASFV infection-induced apoptosis. By inhibiting apoptosis, the virus avoids being exposed to the immune system by host cells, thus facilitating immune escape and viral proliferation (Wang Z. et al., 2022). In addition, studies of A224L and A179L proteins have demonstrated that apoptosis and the survival of ASFV-infected host cells are tightly regulated processes (Figure 7B). Apoptotic pathways in host cells proceed primarily through the extrinsic apoptotic pathway (EAP), also known as the death receptor pathway. The alternative pathway is the intrinsic apoptotic pathway, also known as the mitochondrial apoptotic pathway. Further, analysis of the structure, function, and mechanism of action for these proteins may provide for an understanding of viral proliferation and evasion of immune responses (Dixon et al., 2017).

### 3.6. Proteins involved in ASFV assembly

ASFV contains an envelope, capsid, inner capsule membrane, core shell, and inner core (Wang et al., 2019; Andrés et al., 2020). These five components are assembled into a whole virion. In the assembly process, the components are connected and supported by each other, with structural proteins playing a key role. Some non-structural proteins interact with structural proteins to promote protein structure formation and as such are equally important.

#### 3.6.1. p72 and its molecular chaperone pB602L

**Figure 8:**
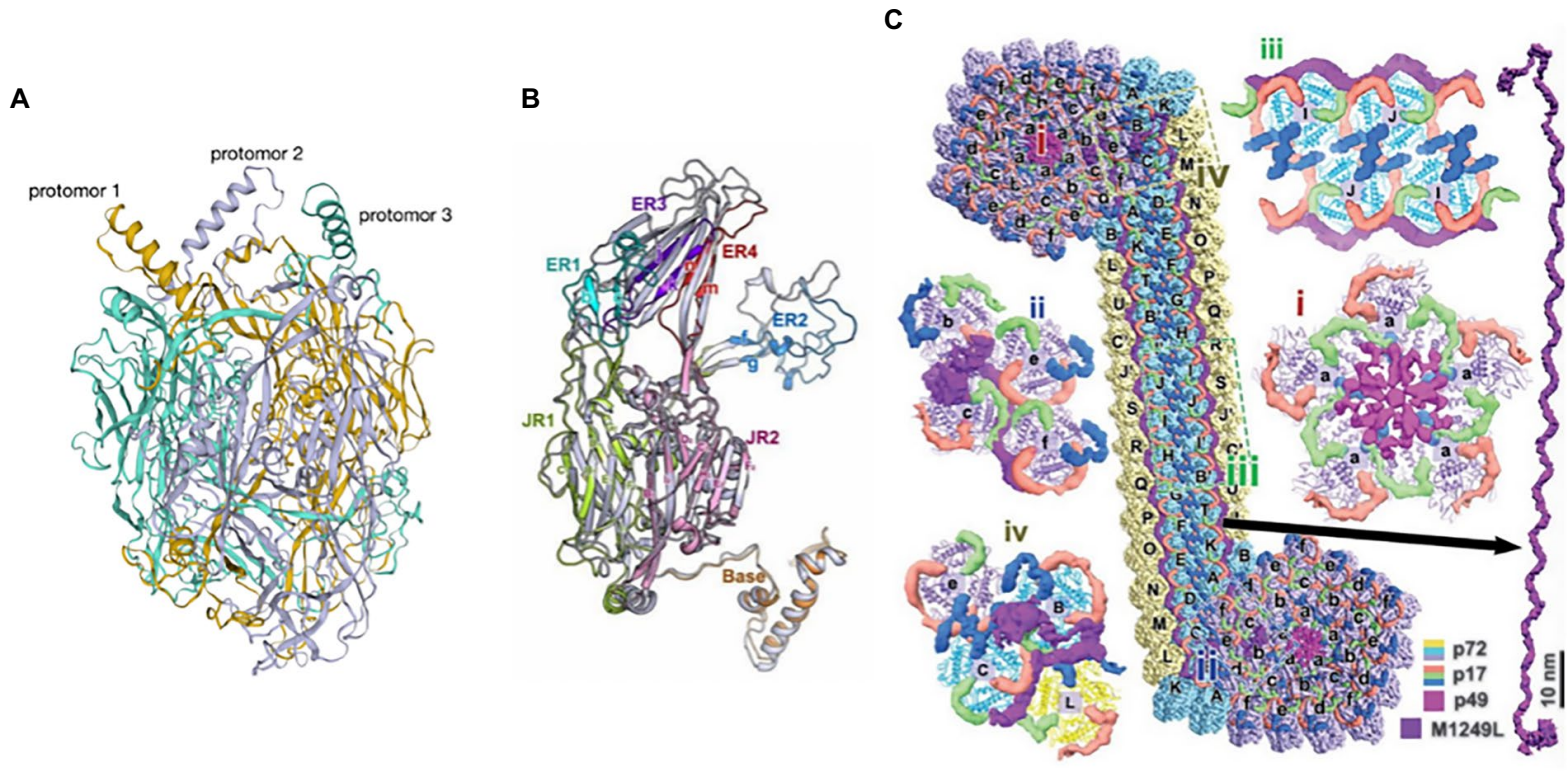
**(A)** The trimeric structure of the p72 protein (Wang et al., 2019). Deep yellow: protomer 1; lavender: protomer 2; cyan: protomer 3. **(B)** Diagram of the p72 monomer. The domains base, JR1 (jelly roll 1), JR2 (jelly roll 2), and ER1 to ER4 are shown (Wang et al., 2019). **(C)** Diagram of the interaction of p72 with H240R, M1249L, p17, and p49 (Wang et al., 2019).

p72 is one of the key protective antigens recognized by the immune system in response to ASFV and also the ASFV major capsid protein (Borca et al., 1994; Escribano et al., 2013). The ASFV capsid is constructed of pseudo-hexameric capsomers and pentameric capsomers. Each pseudo-hexameric capsomer is composed of three p72 molecules, and each pentameric capsomer is composed of five penton proteins (H240R). p72 forms a homotrimer (Figure 8A), with each monomer (Figure 8B) adopting a double jelly-roll structure that makes up pseudo-hexameric capsomers (p72 capsomers; Wang et al., 2019; Meng et al., 2022). ASFV capsid assembly is a gradual process involving p72. First, the penton complex docks with the inner membrane, where it recruits p72 capsomers, which form the penton core and initiate assembly. The skeleton unit M1249L then attaches to the penton core, and p72 capsomers and p17 contribute to the formation of zippers. The zippers connect neighboring penton cores and gradually construct a polyhedral framework. Finally, accompanying the formation of the polyhedral framework, p72 capsomers fill in the trisymmetrons and complete the capsid assembly (Wang et al., 2019; Meng et al., 2022). p72 may have potential neutralizing epitopes. Exposed region 1 (ER1) and ER2 form the crown of the p72 capsomer orienting toward the outside of the capsid, which may contribute to a conformational epitope. The β strands of ER3 and ER4 constitute a four-stranded β-sheet that shapes the head of the p72 capsomer and may be another conformational epitope. ER3 may link these two conformational epitopes, and these four ERs probably define the neutralizing epitopes (Andrés et al., 2020).

p72 also has a molecular chaperone, pB602L, which promotes correct folding of p72. The viral assembly process is severely altered in the absence of pB602L, with the generation of aberrant “zipper-like” structures instead of icosahedral virus particles. Further, repression of pB602L synthesis affects the proteolytic processing of polyproteins, pp220 and pp62, and reduces the expression level of p72 (Epifano et al., 2006). However, the mechanism through which pB602L affects p72 is unclear, and this interaction may be direct. Understanding the structure and function of pB602L may provide valuable insight into the structure of p72.

#### 3.6.2. Interaction of p72 with H240R, M1249L, p17, and p49

H240R is a capsid protein of ASFV that is a penton protein found in the cytoplasm. H240R as an uncharacterized but essential virion protein that exhibits a single jelly roll and a globular cap that is different from, yet homologous to p72 (Wang et al., 2019; Andrés et al., 2020). H240R is encapsulated by a p72 shell that fills the pentameric capsomer and forms an apex to facilitate the assembly of the entire capsid. However, H240R is not involved in viral attachment or entry into pulmonary alveolar macrophages (PAMs) (Zhou et al., 2022). H240R, p17, p49, and M1249L form a complicated network immediately below the outer capsid shell, stabilizing the whole capsid (Figure 8C; Wang et al., 2019; Andrés et al., 2020).

M1249L is a skeleton protein with multiple helices and a fiber-like configuration with two terminal lobes, with the capacity to fix one pentasymmetron and link two neighboring pentasymmetrons. M1249L extensively interacts with p17 and p72 capsomers to form a rigid zipper structure, facilitating the formation of capsids (Wang et al., 2019).

p17 is encoded by the D117L gene and is a major structural transmembrane protein localized in the capsid and inner lipid envelope (Muñoz and Tabarés, 2022; Zheng et al., 2022). p17 is an essential and highly abundant protein required for the assembly of capsids and icosahedral morphogenesis (Xia et al., 2020). p17 is closely associated with the base domain of p72, with three copies of p17 encircling each p72 capsomer within the inner capsid shell, which firmly anchors p72 capsomers on the inner membrane, ensuring the stabilization of capsids (Andrés et al., 2020). Recently, p17 was shown to inhibit cell proliferation through ER stress and ROS-mediated cell cycle arrest, which may implicate p17 in ASF pathogenesis. p17 may also inhibit the cGAS-STING pathway through its interaction with STING and interference in the recruitment of TBK1 and IKKϵ, which would inhibit the IFN response and would promote immune evasion (Xia et al., 2020; Zheng et al., 2022).

p49 is encoded by the ASFV B438L gene, forms the outer capsid with p72, is located in close proximity to the capsid vertices, and is required for the formation of the capsid vertices (Alejo et al., 2018). P49 associates with the membrane where it mediates the docking of the penton complex to the inner membrane, recruiting capsomers to form the penton core, which initiates assembly (Wang et al., 2019). p49 is not involved in particle transportation from the virus assembly site to the plasma membrane (Epifano et al., 2006).

In summary, p72 capsomers are insufficient to assemble higher-order structures through only the three known and distinct assembly patterns: “head to back,” “head to head,” and “back to back” (Wang et al., 2019). H240R, M1249L, p17, and p49 are needed to build the networks that trigger the assembly of the whole capsid. But as the major capsid protein, p72 constitutes most of the outer capsid and is one of the key immune protective antigens of ASFV. The analysis of p72 structure will provide a new reference for the design of antigenic epitope-based vaccines in the future.

## 4. Discussion

ASFV has a complex structure comprised numerous proteins. The function and mechanism of action for these proteins are not well described, which has made difficult the development of an efficient and effective ASF vaccine. Thus, it is necessary to investigate the structure and function of ASFV proteins, as a means by which to understand the infectious mechanisms of the virus. However, significant progress has been made in understanding the structure and function of ASFV proteins. For example, the crystal structure of the major capsid protein, p72, has been solved, thus demonstrating the structure of its homologous trimer and the interaction with H240R, M1249L, p17, and p49 proteins, providing a deeper understanding of the assembly of ASFV in host cells after infection. ASFV has a complex structure, with not only a capsid, but also a capsule, inner membrane, core shell, and genome. Proteins within these structures have specific functions, such as A179L and A224L that inhibit the apoptosis of host cells, and g5Rp that promotes the translation of virus mRNA within host cells. pS273R catalyzes the maturation of pp220 and pp62 multiprotein precursors into the core shell proteins p5, p34, p14, p37, p150, and p15, p35, and p8 that are involved in ASFV. Vaccines based on structural design can improve the antigenicity and immune protective qualities of vaccines (Nuñez Castrejon et al., 2022; Thomas and Abraham, 2022), but ASFV proteins are numerous, with more than 150–200 proteins produced during infection. At present, only a small number of ASFV protein structures are known and the function of most of these proteins is still unknown. Resolution of the structure and function of ASFV proteins will provide a clearer understanding of the interaction between virus and host, and the mechanism of ASFV replication and transmission. Further, this understanding will allow for convenient screening for protective antigens, analysis of neutralization epitopes, and the design of more targeted and effective ASF vaccines.

## Author contributions

SY and WL conceived and designed the study. SY wrote the first draft of the manuscript. SY, WL, CM, and GZ revised the manuscript. SY and CM made the diagram. JS and HC performed the funding acquisition. All authors contributed to the article and approved the submitted version.

## Funding

This work was financially supported by the National Key Research and Development Program of China (2021YFD11801402).

## Conflict of interest

The authors declare that the research was conducted in the absence of any commercial or financial relationships that could be construed as a potential conflict of interest.

## Publisher’s note

All claims expressed in this article are solely those of the authors and do not necessarily represent those of their affiliated organizations, or those of the publisher, the editors and the reviewers. Any product that may be evaluated in this article, or claim that may be made by its manufacturer, is not guaranteed or endorsed by the publisher.

## References

[ref1] Alejo A. Matamoros T. Guerra M. Andrés G. (2018). A proteomic atlas of the African swine fever virus particle. J. Virol. 92:e01293–18. doi: 10.1128/JVI.01293-18, PMID: 30185597 PMC6232493

[ref2] Alonso C. Miskin J. Hernáez B. Fernandez-Zapatero P. Soto L. Cantó C.. (2001). African swine fever virus protein p54 interacts with the microtubular motor complex through direct binding to light-chain dynein. J. Virol. 75, 9819–9827. doi: 10.1128/JVI.75.20.9819-9827.2001, PMID: 11559815 PMC114554

[ref3] Andrés G.´. Alejo A.´. Salas J.´. Salas M.´. L. (2002a). African swine fever virus polyproteins pp220 and pp62 assemble into the core shell. J. Virol. 76, 12473–12482. doi: 10.1128/JVI.76.24.12473-12482.2002, PMID: 12438573 PMC136718

[ref4] Andrés G. Charro D. Matamoros T. Dillard R. S. Abrescia N. G. A. (2020). The cryo-EM structure of African swine fever virus unravels a unique architecture comprising two icosahedral protein capsids and two lipoprotein membranes. J. Biol. Chem. 295, 1–12. doi: 10.1074/jbc.AC119.011196, PMID: 31649031 PMC6952596

[ref5] Andrés G.´. García-Escudero R.´. Salas M.´. L. Rodríguez J. M. (2002b). Repression of African swine fever virus polyprotein pp220-encoding gene leads to the assembly of icosahedral core-less particles. J. Virol. 76, 2654–2666. doi: 10.1128/JVI.76.6.2654-2666.2002, PMID: 11861832 PMC135994

[ref6] Báez-Santos Y. M. St John S. E. Mesecar A. D. (2015). The SARS-coronavirus papain-like protease: structure, function and inhibition by designed antiviral compounds. Antiviral Res. 115, 21–38. doi: 10.1016/j.antiviral.2014.12.015, PMID: 25554382 PMC5896749

[ref7] Banjara S. Caria S. Dixon L. K. Hinds M. G. Kvansakul M. (2017). Structural insight into African swine fever virus A179L-mediated inhibition of apoptosis. J. Virol. 91:e02228. doi: 10.1128/JVI.02228-16, PMID: 28053104 PMC5331815

[ref8] Banjara S. Shimmon G. L. Dixon L. K. Netherton C. L. Hinds M. G. Kvansakul M. (2019). Crystal structure of African swine fever virus A179L with the autophagy regulator Beclin. Viruses 11:789. doi: 10.3390/v11090789, PMID: 31461953 PMC6784060

[ref9] Barber C. Netherton C. Goatley L. Moon A. Goodbourn S. Dixon L. (2017). Identification of residues within the African swine fever virus DP71L protein required for dephosphorylation of translation initiation factor eIF2α and inhibiting activation of pro-apoptotic CHOP. Virology 504, 107–113. doi: 10.1016/j.virol.2017.02.002, PMID: 28189088 PMC5346070

[ref10] Barrado-Gil L. del Puerto A. Muñoz-Moreno R. Galindo I. Cuesta-Geijo M. Á. Urquiza J.. (2020). African swine fever virus ubiquitin-conjugating enzyme interacts with host translation machinery to regulate the host protein synthesis. Front. Microbiol. 11:622907. doi: 10.3389/fmicb.2020.622907, PMID: 33384682 PMC7771050

[ref11] Borca M. V. Irusta P. Carrillo C. Afonso C. L. Burrage T. Rock D. L. (1994). African swine fever virus structural protein p72 contains a conformational neutralizing epitope. Virology 201, 413–418. doi: 10.1006/viro.1994.1311, PMID: 7514322

[ref12] Brun A. Rivas C. Esteban M. Escribano J. M. Alonso C. (1996). African swine fever virus gene A179L, a viral homologue of bcl-2, protects cells from programmed cell death. Virology 225, 227–230. doi: 10.1006/viro.1996.0592, PMID: 8918551

[ref13] Cackett G. Matelska D. Sýkora M. Portugal R. Malecki M. Bähler J.. (2020). The African swine fever virus Transcriptome. J. Virol. 94:e00119–20. doi: 10.1128/JVI.00119-20, PMID: 32075923 PMC7163114

[ref14] Cadenas-Fernández E. Ito S. Aguilar-Vega C. Sánchez-Vizcaíno J. M. Bosch J. (2022). The role of the wild boar spreading African swine fever virus in Asia: another underestimated problem. Front. Vet. Sci. 9:844209. doi: 10.3389/fvets.2022.844209, PMID: 35573420 PMC9093143

[ref15] Cartwright J. L. Safrany S. T. Dixon L. K. Darzynkiewicz E. Stepinski J. Burke R.. (2002). The g5R (D250) gene of African swine fever virus encodes a Nudix hydrolase that preferentially degrades diphosphoinositol polyphosphates. J. Virol. 76, 1415–1421. doi: 10.1128/JVI.76.3.1415-1421.2002, PMID: 11773415 PMC135849

[ref16] Coelho J. Leitão A. (2020). The African swine fever virus (ASFV) topoisomerase II as a target for viral prevention and control. Vaccine (Basel). 8:312. doi: 10.3390/vaccines8020312 PMC7350233 32560397

[ref17] Coelho J. Martins C. Ferreira F. Leitão A. (2015). African swine fever virus ORF P1192R codes for a functional type II DNA topoisomerase. Virology 474, 82–93. doi: 10.1016/j.virol.2014.10.034, PMID: 25463606

[ref18] Cong P. Shuman S. (1995). Mutational analysis of mRNA capping enzyme identifies amino acids involved in GTP binding, enzyme-guanylate formation, and GMP transfer to RNA. Mol. Cell. Biol. 15, 6222–6231. doi: 10.1128/MCB.15.11.6222, PMID: 7565775 PMC230874

[ref19] Constantinou A. Mehta R. Runyan C. Rao K. Vaughan A. Moon R. (1995). Flavonoids as DNA topoisomerase antagonists and poisons: structure-activity relationships. J. Nat. Prod. 58, 217–225. doi: 10.1021/np50116a009, PMID: 7769390

[ref20] Cooper T. L. Smith D. Gonzales M. J. C. Maghanay M. T. Sanderson S. Cornejo M. R. J. C.. (2021). Beyond numbers: determining the socioeconomic and livelihood impacts of African swine fever and its control in the Philippines. Front. Vet. Sci. 8:734236. doi: 10.3389/fvets.2021.734236 35224068 PMC8866713

[ref21] Costard S. Wieland B. de Glanville W. Jori F. Rowlands R. Vosloo W.. (2009). African swine fever: how can global spread be prevented? Philos. Trans. R. Soc. Lond. B Biol. Sci. 364, 2683–2696. doi: 10.1098/rstb.2009.0098, PMID: 19687038 PMC2865084

[ref22] de Rosa M. Zacarias S. Athanasiadis A. (2013). Structural basis for Z-DNA binding and stabilization by the zebrafish Z-DNA dependent protein kinase PKZ. Nucleic Acids Res. 41, 9924–9933. doi: 10.1093/nar/gkt743, PMID: 23975196 PMC3834819

[ref23] Decroly E. Ferron F. Lescar J. Canard B. (2011). Conventional and unconventional mechanisms for capping viral mRNA. Nat. Rev. Microbiol. 10, 51–65. doi: 10.1038/nrmicro2675, PMID: 22138959 PMC7097100

[ref24] Deveraux Q. L. Takahashi R. Salvesen G. S. Reed J. C. (1997). X-linked IAP is a direct inhibitor of cell-death proteases. Nature 388, 300–304. doi: 10.1038/40901 9230442

[ref25] Dixon L. K. Chapman D. A. G. Netherton C. L. Upton C. (2013). African swine fever virus replication and genomics. Virus Res. 173, 3–14. doi: 10.1016/j.virusres.2012.10.020 23142553

[ref26] Dixon L. K. Sánchez-Cordón P. J. Galindo I. Alonso. (2017). Investigations of pro- and anti-apoptotic factors affecting African swine fever virus replication and pathogenesis. Viruses 9:241. doi: 10.3390/v9090241, PMID: 28841179 PMC5618007

[ref27] Du X. Gao Z. Q. Geng Z. Dong Y. H. Zhang. (2020). Structure and biochemical characteristic of the Methyltransferase (MTase) domain of RNA capping enzyme from African swine fever virus. J. Virol. 95:e02029–20. doi: 10.1128/JVI.02029-20 PMC8092831 33268516

[ref28] Epifano C. Krijnse-Locker J. Salas M.´. L. Rodríguez J. M. Salas J.´. (2006). The African swine fever virus nonstructural protein pB602L is required for formation of the icosahedral capsid of the virus particle. J. Virol. 80, 12260–12270. doi: 10.1128/JVI.01323-06, PMID: 17035321 PMC1676282

[ref29] Escribano J. M. Galindo I. Alonso C. (2013). Antibody-mediated neutralization of African swine fever virus: myths and facts. Virus Res. 173, 101–109. doi: 10.1016/j.virusres.2012.10.012, PMID: 23159730

[ref30] Eulálio A. Nunes-Correia I. Carvalho A. L. Faro C. Citovsky V. Simões S.. (2004). Two African swine fever virus proteins derived from a common precursor exhibit different nucleocytoplasmic transport activities. J. Virol. 78, 9731–9739. doi: 10.1128/JVI.78.18.9731-9739.2004, PMID: 15331706 PMC514976

[ref31] Eulálio A. Nunes-Correia I. Salas J. Salas M. L. Simões S. Pedroso de Lima M. C. (2007). African swine fever virus p37 structural protein is localized in nuclear foci containing the viral DNA at early post-infection times. Virus Res. 130, 18–27. doi: 10.1016/j.virusres.2007.05.009, PMID: 17580096

[ref32] Freitas F. B. Frouco G. Martins C. Ferreira F. (2018). African swine fever virus encodes for an E2-ubiquitin conjugating enzyme that is mono- and di-ubiquitinated and required for viral replication cycle. Sci. Rep. 8:3471. doi: 10.1038/s41598-018-21872-2, PMID: 29472632 PMC5823848

[ref33] Freitas F. B. Frouco G. Martins C. Ferreira F. (2019). The QP509L and Q706L superfamily II RNA helicases of African swine fever virus are required for viral replication, having non-redundant activities. Emerg. Microbes Infect. 8, 291–302. doi: 10.1080/22221751.2019.1578624, PMID: 30866783 PMC6455146

[ref34] Frouco G. Freitas F. B. Coelho J. Leitão A. Martins C. Ferreira F. (2017). DNA-binding properties of African swine fever virus pA104R, a histone-like protein involved in viral replication and transcription. J. Virol. 91:e02498. doi: 10.1128/JVI.02498-16, PMID: 28381576 PMC5446646

[ref35] Galindo I. Cuesta-Geijo M. A. Hlavova K. Muñoz-Moreno R. Barrado-Gil L. Dominguez J.. (2015). African swine fever virus infects macrophages, the natural host cells, via clathrin- and cholesterol-dependent endocytosis. Virus Res. 200, 45–55. doi: 10.1016/j.virusres.2015.01.022, PMID: 25662020

[ref36] Galindo I. Hernaez B. Díaz-Gil G. Escribano J. M. Alonso C. (2008). A179L, a viral Bcl-2 homologue, targets the core Bcl-2 apoptotic machinery and its upstream BH3 activators with selective binding restrictions for bid and Noxa. Virology 375, 561–572. doi: 10.1016/j.virol.2008.01.050, PMID: 18329683 PMC2572728

[ref37] Gaudreault N. N. Madden D. W. Wilson W. C. Trujillo J. D. Richt J. A. (2020). African swine fever virus: An emerging DNA Arbovirus. Front. Vet. Sci. 7:215. doi: 10.3389/fvets.2020.00215, PMID: 32478103 PMC7237725

[ref38] Geng R. Sun Y. Li R. Yang J. Ma H. Qiao Z.. (2022). Development of a p72 trimer-based colloidal gold strip for detection of antibodies against African swine fever virus. Appl. Microbiol. Biotechnol. 106, 2703–2714. doi: 10.1007/s00253-022-11851-z, PMID: 35291024 PMC8923092

[ref39] Goatley L. C. Dixon L. K. (2011). Processing and localization of the african swine fever virus CD2v transmembrane protein. J. Virol. 85, 3294–3305. doi: 10.1128/JVI.01994-10, PMID: 21248037 PMC3067853

[ref40] Grosche P. Sirockin F. Mac Sweeney A. Ramage P. Erbel P. Melkko S.. (2015). Structure-based design and optimization of potent inhibitors of the adenoviral protease. Bioorg. Med. Chem. Lett. 25, 438–443. doi: 10.1016/j.bmcl.2014.12.057, PMID: 25571794

[ref41] Guo F. Shi Y. Yang M. Guo Y. Shen Z. Li M.. (2021). The structural basis of African swine fever virus core shell protein p15 binding to DNA. FASEB J. 35:e21350. doi: 10.1096/fj.202002145R, PMID: 33629764

[ref42] Ha S. C. Choi J. Hwang H. Y. Rich A. Kim Y. G. Kim K. K. (2009). The structures of non-CG-repeat Z-DNAs co-crystallized with the Z-DNA-binding domain, hZ alpha(ADAR1). Nucleic Acids Res. 37, 629–637. doi: 10.1093/nar/gkn976, PMID: 19074195 PMC2632926

[ref43] Ha S. C. Kim D. Hwang H. Y. Rich A. Kim Y. G. Kim K. K. (2008). The crystal structure of the second Z-DNA binding domain of human DAI (ZBP1) in complex with Z-DNA reveals an unusual binding mode to Z-DNA. Proc. Natl. Acad. Sci. U. S. A. 105, 20671–20676. doi: 10.1073/pnas.0810463106, PMID: 19095800 PMC2634953

[ref44] Hakizimana J. N. Nyabongo L. Ntirandekura J. B. Yona C. Ntakirutimana D. Kamana O.. (2020). Genetic analysis of African swine fever virus from the 2018 outbreak in south-eastern Burundi. Front. Vet. Sci. 7:578474. doi: 10.3389/fvets.2020.578474, PMID: 33251264 PMC7674587

[ref45] Hakobyan A. Galindo I. Nañez A. Arabyan E. Karalyan Z. Chistov A. A.. (2018). Rigid amphipathic fusion inhibitors demonstrate antiviral activity against African swine fever virus. J. Gen. Virol. 99, 148–156. doi: 10.1099/jgv.0.000991, PMID: 29235978

[ref46] Harvey A. J. Soliman H. Kaiser W. J. Miller L. K. (1997). Anti- and pro-apoptotic activities of baculovirus and drosophila IAPs in an insect cell line. Cell Death Differ. 4, 733–744. doi: 10.1038/sj.cdd.4400294, PMID: 16465286

[ref47] Hernáez B. Díaz-Gil G. García-Gallo M. Ignacio Quetglas J. Rodríguez-Crespo I. Dixon L.. (2004). The African swine fever virus dynein-binding protein p54 induces infected cell apoptosis. FEBS Lett. 569, 224–228. doi: 10.1016/j.febslet.2004.06.001, PMID: 15225638

[ref48] Hernáez B. Guerra M. Salas M. L. Andrés G. (2016). African swine fever virus undergoes outer envelope disruption, capsid disassembly and inner envelope fusion before Core release from multivesicular endosomes. PLoS Pathog. 12:e1005595. doi: 10.1371/journal.ppat.1005595, PMID: 27110717 PMC4844166

[ref49] Hernáez B. Tarragó T. Giralt E. Escribano J. M. Alonso C. (2010). Small peptide inhibitors disrupt a high-affinity interaction between cytoplasmic dynein and a viral cargo protein. J. Virol. 84, 10792–10801. doi: 10.1128/JVI.01168-10, PMID: 20686048 PMC2950572

[ref50] Iscaro C. Cambiotti V. Bessi O. Pacelli F. Ruocco L. Feliziani F. (2022). *Analysis of surveillance and prevention plan for African swine fever in Italy in 2020.* Vet. Med. Sci. 8, 1502–1508. doi: 10.1002/vms3.824, PMID: 35675914 PMC9297771

[ref51] Istrate C. Marques J. Bule P. Correia S. Aires-da-Silva F. Duarte M.. (2022). In Silico characterization of African swine fever virus nucleoprotein p10 interaction with DNA. Viruses 14:2348. doi: 10.3390/v14112348, PMID: 36366446 PMC9694697

[ref52] Jia N. Ou Y. Pejsak Z. Zhang Y. Zhang J. (2017). Roles of African swine fever virus structural proteins in viral infection. J. Vet. Res. 61, 135–143. doi: 10.1515/jvetres-2017-0017, PMID: 29978065 PMC5894393

[ref53] Kinyanyi D. Obiero G. Obiero G. F. O. Amwayi P. Mwaniki S. Wamalwa M. (2018). In silico structural and functional prediction of African swine fever virus protein-B263R reveals features of a TATA-binding protein. PeerJ 6:e4396. doi: 10.7717/peerj.4396, PMID: 29492339 PMC5825884

[ref54] Kouloulia S. Hallin E. I. Simbriger K. Amorim I. S. Lach G. Amvrosiadis T.. (2019). Raptor-mediated proteasomal degradation of Deamidated 4E-BP2 regulates postnatal neuronal translation and NF-κB activity. Cell Rep. 29, 3620–3635.e7. doi: 10.1016/j.celrep.2019.11.023, PMID: 31825840 PMC6915327

[ref55] Kvansakul M. Hinds M. G. (2013). Structural biology of the Bcl-2 family and its mimicry by viral proteins. Cell Death Dis. 4:e909. doi: 10.1038/cddis.2013.436, PMID: 24201808 PMC3847314

[ref56] Lee A. R. Park C. J. Cheong H. K. Ryu K. S. Park J. W. Kwon M. Y.. (2016). Solution structure of the Z-DNA binding domain of PKR-like protein kinase from Carassius auratus and quantitative analyses of the intermediate complex during B-Z transition. Nucleic Acids Res. 44, 2936–2948. doi: 10.1093/nar/gkw025, PMID: 26792893 PMC4824103

[ref57] Li G. Liu X. Yang M. Zhang G. Wang Z. Guo K.. (2020). Crystal structure of African swine fever virus pS273R protease and implications for inhibitor design. J. Virol. 94:e02125. doi: 10.1128/JVI.02125-19, PMID: 32075933 PMC7199414

[ref58] Li D. Wu P. Liu H. Feng T. Yang W. Ru Y.. (2022). A QP509L/QP383R-deleted African swine fever virus is highly attenuated in swine but does not confer protection against parental virus challenge. J. Virol. 96:e0150021. doi: 10.1128/JVI.01500-21, PMID: 34613824 PMC8754219

[ref59] Liu S. Luo Y. Wang Y. Li S. Zhao Z. Bi Y.. (2019). Cryo-EM structure of the African swine fever virus. Cell Host Microbe 26, 836–843.e3. doi: 10.1016/j.chom.2019.11.004, PMID: 31787524

[ref60] Liu R. Sun Y. Chai Y. Li S. Li S. Wang L.. (2020). The structural basis of African swine fever virus pA104R binding to DNA and its inhibition by stilbene derivatives. Proc. Natl. Acad. Sci. U. S. A. 117, 11000–11009. doi: 10.1073/pnas.1922523117, PMID: 32358196 PMC7245070

[ref61] Liu Y. Zhang X. Qi W. Yang Y. Liu Z. An T.. (2021). Prevention and control strategies of African swine fever and Progress on pig farm repopulation in China. Viruses 13:2552. doi: 10.3390/v13122552, PMID: 34960821 PMC8704102

[ref62] Mac Sweeney A. Grosche P. Ellis D. Combrink K. Erbel P. Hughes N.. (2014). Discovery and structure-based optimization of adenain inhibitors. ACS Med. Chem. Lett. 5, 937–941. doi: 10.1021/ml500224t, PMID: 25147618 PMC4137446

[ref63] Marciniak S. J. Yun C. Y. Oyadomari S. Novoa I. Zhang Y. Jungreis R.. (2004). CHOP induces death by promoting protein synthesis and oxidation in the stressed endoplasmic reticulum. Genes Dev. 18, 3066–3077. doi: 10.1101/gad.1250704, PMID: 15601821 PMC535917

[ref64] Matamoros T. Alejo A. Rodríguez J. M. Hernáez B. Guerra M. Fraile-Ramos A.. (2020). African swine fever virus protein pE199L mediates virus entry by enabling membrane fusion and Core penetration. MBio 11:e00789–20. doi: 10.1128/mBio.00789-20, PMID: 32788374 PMC7439464

[ref65] Mebus C. A. (1988). African swine fever. Adv. Virus Res. 35, 251–269. doi: 10.1016/S0065-3527(08)60714-9 3068966

[ref66] Meng K. Zhang Y. Liu Q. Huyan Y. Zhu W. Xiang Y.. (2022). Structural design and assessing of Recombinantly expressed African swine fever virus p72 Trimer in Saccharomyces cerevisiae. Front. Microbiol. 13:802098. doi: 10.3389/fmicb.2022.802098, PMID: 35774459 PMC9239254

[ref67] Mercer J. Schelhaas M. Helenius A. (2010). Virus entry by endocytosis. Annu. Rev. Biochem. 79, 803–833. doi: 10.1146/annurev-biochem-060208-104626 20196649

[ref68] Mighell E. Ward M. P. (2021). African swine fever spread across Asia, 2018-2019. Transbound. Emerg. Dis. 68, 2722–2732. doi: 10.1111/tbed.14039, PMID: 33599077

[ref69] Moss B. (2012). Poxvirus cell entry: how many proteins does it take? Viruses 4, 688–707. doi: 10.3390/v4050688, PMID: 22754644 PMC3386626

[ref70] Mottola C. Freitas F. B. Simões M. Martins C. Leitão A. Ferreira F. (2013). In vitro antiviral activity of fluoroquinolones against African swine fever virus. Vet. Microbiol. 165, 86–94. doi: 10.1016/j.vetmic.2013.01.018, PMID: 23415476

[ref71] Muñoz A. L. Tabarés E. (2022). Characteristics of the major structural proteins of African swine fever virus: role as antigens in the induction of neutralizing antibodies. A review. Virology 571, 46–51. doi: 10.1016/j.virol.2022.04.001, PMID: 35500304

[ref72] Nitiss J. L. (2009). DNA topoisomerase II and its growing repertoire of biological functions. Nat. Rev. Cancer 9, 327–337. doi: 10.1038/nrc2608, PMID: 19377505 PMC2730144

[ref73] Nogal, M. L., González de Buitrago, G., Rodríguez, C., Cubelos, B., Carrascosa, A. L., Salas M. L.. (2001). African swine fever virus IAP homologue inhibits caspase activation and promotes cell survival in mammalian cells. J. Virol. 75, 2535–2543. doi: 10.1128/JVI.75.6.2535-2543.2001, PMID: 11222676 PMC115875

[ref74] Nunes-Correia I. Rodríguez J. M. Eulálio A. Carvalho A. L. Citovsky V. Simões S.. (2008). African swine fever virus p10 protein exhibits nuclear import capacity and accumulates in the nucleus during viral infection. Vet. Microbiol. 130, 47–59. doi: 10.1016/j.vetmic.2007.12.010, PMID: 18243588

[ref75] Nuñez Castrejon A. M. O’Rourke S. M. Kauvar L. M. DuBois R. M. (2022). Structure-based design and antigenic validation of respiratory syncytial virus G Immunogens. J. Virol. 96:e0220121. doi: 10.1128/jvi.02201-21, PMID: 35266806 PMC9006937

[ref76] O’Neill X. White A. Ruiz-Fons F. Gortázar C. (2020). Modelling the transmission and persistence of African swine fever in wild boar in contrasting European scenarios. Sci. Rep. 10:5895. doi: 10.1038/s41598-020-62736-y, PMID: 32246098 PMC7125206

[ref77] Parrish S. Hurchalla M. Liu S. W. Moss B. (2009). The African swine fever virus g5R protein possesses mRNA decapping activity. Virology 393, 177–182. doi: 10.1016/j.virol.2009.07.026, PMID: 19695654 PMC3392020

[ref78] Pena L. Yàñez R. J. Revilla Y. Viñuela E. Salas M. L. (1993). African swine fever virus guanylyltransferase. Virology 193, 319–328. doi: 10.1006/viro.1993.1128, PMID: 8382399

[ref79] Penrith M. L. Kivaria F. M. (2022). One hundred years of African swine fever in Africa: where have we been, where are we now, where are we going? Transbound. Emerg. Dis. 69, e1179–e1200. doi: 10.1111/tbed.14466, PMID: 35104041

[ref80] Ramirez-Medina E. O’Donnell V. Silva E. Espinoza N. Velazquez-Salinas L. Moran K.. (2022a). Experimental infection of domestic pigs with an African swine fever virus field strain isolated in 2021 from the Dominican Republic. Viruses 14:1090. doi: 10.3390/v14051090, PMID: 35632831 PMC9145207

[ref81] Ramirez-Medina E. Vuono E. A. Pruitt S. Rai A. Espinoza N. Spinard E.. (2022b). Deletion of an African swine fever virus ATP-dependent RNA helicase QP509L from the highly virulent Georgia 2010 strain does not affect replication or virulence. Viruses 14:2548. doi: 10.3390/v14112548, PMID: 36423157 PMC9694930

[ref82] Revilla Y. Cebrián A. Baixerás E. Martínez-A C. Viñuela E. Salas M.´. L. (1997). Inhibition of apoptosis by the African swine fever virus Bcl-2 homologue: role of the BH1 domain. Virology 228, 400–404. doi: 10.1006/viro.1996.8395, PMID: 9123849

[ref83] Revilla Y. Pérez-Núñez D. Richt J. A. (2018). African swine fever virus biology and vaccine approaches. Adv. Virus Res. 100, 41–74. doi: 10.1016/bs.aivir.2017.10.002, PMID: 29551143

[ref84] Rivera J. Abrams C. Hernáez B. Alcázar A. Escribano J.´. M. Dixon L.. (2007). The MyD116 African swine fever virus homologue interacts with the catalytic subunit of protein phosphatase 1 and activates its phosphatase activity. J. Virol. 81, 2923–2929. doi: 10.1128/JVI.02077-06, PMID: 17215279 PMC1865990

[ref85] Rodriguez F. Alcaraz C. Eiras A. Yáñez R. J. Rodriguez J. M. Alonso C.. (1994). Characterization and molecular basis of heterogeneity of the African swine fever virus envelope protein p54. J. Virol. 68, 7244–7252. doi: 10.1128/jvi.68.11.7244-7252.1994, PMID: 7933107 PMC237164

[ref86] Rodríguez J. M. García-Escudero R.´. Salas M.´. L. Andrés G.´. (2004). African swine fever virus structural protein p54 is essential for the recruitment of envelope precursors to assembly sites. J. Virol. 78, 4299–4313. doi: 10.1128/JVI.78.8.4299-4313.2004, PMID: 15047843 PMC374266

[ref87] Rodríguez I. Nogal M.´. L. Redrejo-Rodríguez M. Bustos M. J. Salas M.´. L. (2009). The African swine fever virus virion membrane protein pE248R is required for virus infectivity and an early postentry event. J. Virol. 83, 12290–12300. doi: 10.1128/JVI.01333-09, PMID: 19793823 PMC2786719

[ref88] Rodríguez, C. I., Nogal, M. L., Carrascosa, A. L., Salas, M. L., Fresno, M., and Revilla Y.. (2002). African swine fever virus IAP-like protein induces the activation of nuclear factor kappa B. J. Virol. 76, 3936–3942. doi: 10.1128/JVI.76.8.3936-3942.2002, PMID: 11907233 PMC136102

[ref89] Romagnoli A. D’Agostino M. Ardiccioni C. Maracci C. Motta S. la Teana A.. (2021). Control of the eIF4E activity: structural insights and pharmacological implications. Cell. Mol. Life Sci. 78, 6869–6885. doi: 10.1007/s00018-021-03938-z, PMID: 34541613 PMC8558276

[ref90] Sánchez E. G. Quintas A. Nogal M. Castelló A. Revilla Y. (2013). African swine fever virus controls the host transcription and cellular machinery of protein synthesis. Virus Res. 173, 58–75. doi: 10.1016/j.virusres.2012.10.025, PMID: 23154157

[ref91] Sauter-Louis C. Conraths F. J. Probst C. Blohm U. Schulz K. Sehl J.. (2021). African swine fever in wild boar in Europe-A review. Viruses 13:1717. doi: 10.3390/v13091717, PMID: 34578300 PMC8472013

[ref92] Shimmon G. L. Hui J. Y. K. Wileman T. E. Netherton C. L. (2021). Autophagy impairment by African swine fever virus. J. Gen. Virol. 102:001637. doi: 10.1099/jgv.0.001637, PMID: 34406116 PMC8513644

[ref93] Shuman S. (2001). Structure, mechanism, and evolution of the mRNA capping apparatus. Prog. Nucleic Acid Res. Mol. Biol. 66, 1–40. doi: 10.1016/s0079-6603(00)66025-7 11051760

[ref94] Simón-Mateo C. Andrés G. Almazán F. Viñuela E. (1997). Proteolytic processing in African swine fever virus: evidence for a new structural polyprotein, pp62. J. Virol. 71, 5799–5804. doi: 10.1128/jvi.71.8.5799-5804.1997, PMID: 9223468 PMC191834

[ref95] Sun L. Miao Y. Wang Z. Chen H. Dong P. Zhang H.. (2022). Structural insight into African swine fever virus I73R protein reveals it as a Z-DNA binding protein. Transbound. Emerg. Dis. 69, e1923–e1935. doi: 10.1111/tbed.14527, PMID: 35312168

[ref96] Sun H. Niu Q. Yang J. Zhao Y. Tian Z. Fan J.. (2021). Transcriptome profiling reveals features of immune response and metabolism of acutely infected, dead and asymptomatic infection of African swine fever virus in pigs. Front. Immunol. 12:808545. doi: 10.3389/fimmu.2021.808545, PMID: 34975923 PMC8714921

[ref97] Tao D. Sun D. Liu Y. Wei S. Yang Z. An T.. (2020). One year of African swine fever outbreak in China. Acta Trop. 211:105602. doi: 10.1016/j.actatropica.2020.105602, PMID: 32598922

[ref98] Thomas S. Abraham A. (2022). Progress in the development of structure-based vaccines. Methods Mol. Biol. 2412, 15–33. doi: 10.1007/978-1-0716-1892-9_2, PMID: 34918239

[ref99] Urbano A. C. Ferreira F. (2020). Role of the DNA-binding protein pA104R in ASFV genome packaging and as a novel target for vaccine and drug development. Vaccine (Basel) 8:585. doi: 10.3390/vaccines8040585 PMC7712801 33023005

[ref100] Valdeira M. L. Bernardes C. Cruz B. Geraldes A. (1998). Entry of African swine fever virus into Vero cells and uncoating. Vet. Microbiol. 60, 131–140. doi: 10.1016/S0378-1135(98)00152-7, PMID: 9646445

[ref101] Vergne T. Korennoy F. Combelles L. Gogin A. Pfeiffer D. U. (2016). Modelling African swine fever presence and reported abundance in the Russian Federation using national surveillance data from 2007 to 2014. Spat. Spatiotemporal Epidemiol. 19, 70–77. doi: 10.1016/j.sste.2016.06.002, PMID: 27839582

[ref102] Wang H. L. Chang N. C. Weng Y. H. Yeh T. H. (2013). XLID CUL4B mutants are defective in promoting TSC2 degradation and positively regulating mTOR signaling in neocortical neurons. Biochim. Biophys. Acta 1832, 585–593. doi: 10.1016/j.bbadis.2013.01.010, PMID: 23348097

[ref103] Wang Y. Kang W. Yang W. Zhang J. Li D. Zheng H. (2021). Structure of African swine fever virus and associated molecular mechanisms underlying infection and immunosuppression: A review. Front. Immunol. 12:715582. doi: 10.3389/fimmu.2021.715582, PMID: 34552586 PMC8450572

[ref104] Wang G. Xie M. Wu W. Chen Z. (2021). Structures and functional diversities of ASFV proteins. Viruses 13:2124. doi: 10.3390/v13112124, PMID: 34834930 PMC8619059

[ref01] Wang Z. Ai Q. Huang S. Ou Y. Gao Y. Tong T.. (2022). Immune escape mechanism and vaccine research Progress of African swine fever virus. Vaccines (Basel). 10:344. doi: 10.3390/vaccines10030344, PMID: 35334976 PMC8949402

[ref105] Wang N. Zhao D. Wang J. Zhang Y. Wang M. Gao Y.. (2019). Architecture of African swine fever virus and implications for viral assembly. Science 366, 640–644. doi: 10.1126/science.aaz1439, PMID: 31624094

[ref106] Wang, C., Songyin, Q., Ying, X., Haoyang, Y., Haoxuan, L., Shaoqiang W.. (2022). Development of a blocking ELISA kit for detection of ASFV antibody based on a monoclonal antibody against full length p72. J. AOAC Int. 105, 1428–1436. doi: 10.1093/jaoacint/qsac050 35595230

[ref108] Xia N. Wang H. Liu X. Shao Q. Ao D. Xu Y.. (2020). African swine fever virus structural protein p17 inhibits cell proliferation through ER stress-ROS mediated cell cycle arrest. Viruses 13:21. doi: 10.3390/v13010021, PMID: 33374251 PMC7823474

[ref109] Yang Y. Zhang C. Li X. Li L. Chen Y. Yang X.. (2022). Structural insight into molecular inhibitory mechanism of InsP(6) on African swine fever virus mRNA-Decapping enzyme g5Rp. J. Virol. 96:e0190521. doi: 10.1128/jvi.01905-21, PMID: 35481780 PMC9131872

[ref110] Zhang K. Li S. Liu S. Li S. Qu L. Gao G. F.. (2021). Spatiotemporally orchestrated interactions between viral and cellular proteins involved in the entry of African swine fever virus. Viruses 13:2495. doi: 10.3390/v13122495, PMID: 34960765 PMC8703583

[ref111] Zhang F. Moon A. Childs K. Goodbourn S. Dixon L. K. (2010). The African swine fever virus DP71L protein recruits the protein phosphatase 1 catalytic subunit to dephosphorylate eIF2alpha and inhibits CHOP induction but is dispensable for these activities during virus infection. J. Virol. 84, 10681–10689. doi: 10.1128/JVI.01027-10, PMID: 20702639 PMC2950568

[ref112] Zheng W. Xia N. Zhang J. Cao Q. Jiang S. Luo J.. (2022). African swine fever virus structural protein p17 inhibits cGAS-STING signaling pathway through interacting with STING. Front. Immunol. 13:941579. doi: 10.3389/fimmu.2022.941579, PMID: 35844609 PMC9283692

[ref113] Zhong H. Fan S. du Y. Zhang Y. Zhang A. Jiang D.. (2022). African swine fever virus MGF110-7L induces host cell translation suppression and stress granule formation by activating the PERK/PKR-eIF2α pathway. Microbiol. Spectr. 10:e0328222. doi: 10.1128/spectrum.03282-22, PMID: 36377947 PMC9769596

[ref114] Zhou P. Li L. F. Zhang K. Wang B. Tang L. Li M.. (2022). Deletion of the H240R gene of African swine fever virus decreases infectious progeny virus production due to aberrant Virion morphogenesis and enhances inflammatory cytokine expression in porcine macrophages. J. Virol. 96:e0166721. doi: 10.1128/jvi.01667-21, PMID: 34787458 PMC8826909

